# Effect of *Nerium oleander* (N.O.) Leaves Extract on Serum *hepcidin*, Total Iron, and Infiltration of ED1 Positive Cells in Albino Rat

**DOI:** 10.1155/2013/125671

**Published:** 2013-08-31

**Authors:** Muddasir Hassan Abbasi, Sana Fatima, Naila Naz, Ihtzaz A. Malik, Nadeem Sheikh

**Affiliations:** ^1^Department of Zoology, Government College of Science, Wahdat Road, Lahore 54590, Pakistan; ^2^Cell and Molecular Biology Lab, Department of Zoology, University of the Punjab, Lahore 54590, Pakistan; ^3^University of Health Sciences, Lahore 54590, Pakistan; ^4^Department of Internal Medicine, University of Goettingen, 37075 Goettingen, Germany

## Abstract

To gain insight into the hepatohistological alterations in noninjured rat liver, *Nerium oleander* (N.O.) leaves extract was injected intramuscularly to induce an acute phase reaction (APR). Histopathological changes were studied after 3, 12, and 24 h time course of sterile muscle abscess. Tissue integrity and any infiltration of inflammatory cells in the liver were investigated by Hematoxylin and Eosin and ED1 peroxidase stainings. The administration of N.O. leaves extract (10 mL/kg) in H & E stained sections showed a general vacuolization of cytoplasm resulting loss of polarity with prominent nucleoli after 3 h of induction. At 12 h, eccentric nuclei were also observed in the sections. Marked infiltration of leucocytes with predominate macrophages was also found after 24 h as seen by ED1 positive staining. In the present study, a possible relationship between serum *hepcidin* and total iron level was also investigated *in vivo*. An early increase of *hepcidin* and total iron level (3 h) with a maximum at 12 h (*P* < 0.01; *P* < 0.001) was observed. These changes indicate that sterile muscle abscess may induce APR resulting in hepatic damage which is evident with the recruitment of inflammatory cells into the organ.

## 1. Introduction 

With iron in being integral part of numerous cellular metabolic activities [1, 2], its homeostasis is controlled by a large group of iron-regulatory proteins, but it excess in the body becomes potentially toxic to the cell because mammals lack a regulatory pathway for its excretion [3]. Erythrocytes besides spleen and liver contain the majority of body iron as a component of hemoglobin and circulate throughout the body for vital redox biological processes. Alterations in iron storage are associated under some pathological conditions, triggering oxidative stress and inflammation [4–7]. Excessive intake of this element in terms of iron-containing medicine and supplements is considered to play a role in the onset of liver cell damage in some cases, cirrhosis of the organ [8, 9], as free iron induces the production of proinflammatory and fibrogenic mediators such as TNF-*α* and transforming growth factor-*β* (TGF-*β*) and nuclear factor-*β* (NF-*Κ*B) activation in hepatic macrophages [10–12].

Inflammation is the major, innate, and complex biological response of the body to stimuli, both exogenous and endogenous or against invading pathogens and infection upon tissue injury. It consists of activation and recruitment of leukocytes and certain plasma proteins at the site of affected tissue to eliminate the causative agent [13–16]. This local inflammatory response is later accompanied by a prominent systemic response known as acute phase response (APR) [17]. Systemic injuries provoke a coordinated change in the hepatic synthesis, hematopoietic profile, and levels of a variety of plasma proteins [18]. The proteins that respond during APR are usually referred to as acute phase proteins (APPs) or acute-phase reactants. Synthesis of the major APPs can increase to 1000-fold over normal levels during acute-phase condition [19], and they have the potential to influence one or another stage of the inflammatory response [20]. Elevated expressions of APPs differ widely from species to species, and their pattern often depends upon sex [21].

The liver plays an important role during injury by modulating immune function, inflammatory processes, and the acute phase response, which are an orchestrated attempt to restore homeostasis [22]. Concentrations of total protein and albumin in the plasma and liver are commonly used parameters to evaluate liver functions. Wide range of metabolic alterations in these proteins and related metabolites is an indication of severe liver injury as it is the principal organ liable for the synthesis of such proteins [23–25]. 

*hepcidin*, a peptide hormone, isolated from plasma ultra-filtrate and named liver-expressed antimicrobial peptide (LEAP-1) is a central regulator of systemic iron balance mainly synthesized by hepatocytes in the liver [26–30]. It regulates intestinal iron absorption [31, 32] as well as maternal fetal iron transport across the placenta [33]. *hepcidin* is a 25-amino acid, 2- to 3-kDa, acute-phase protein [34], whose production is increased during inflammation and in iron-overload conditions [16]. It binds to and initiates degradation of ferroportin-1 (FPN-1), the sole elemental iron exporter in vertebrates [35–38]. Loss of FPN-1 activity prevents mobilization of iron to the bloodstream from intracellular stores in enterocytes and reticuloendothelial macrophages, leading to hypoferremia and anemia, even in the presence of sufficient dietary iron [34, 37, 39, 40]. 

Ethnomedicines have the potential to be both therapeutic and harmful, but still masses of indigenous population rely on these remedies. The medicinal value of the plants lies in the bioactive phytochemicals, but their poisoning may results in toxicological emergencies [41, 42]. Botanical origin, chemical composition, contamination, and degradation of these chemicals affect their performance and efficacy. Almost all plant parts especially the leaves are frequently used for medicinal purposes [43].

*Nerium oleander* (N.O) (Apocynaceae) is an ornamental shrub distributed originally in the Mediterranean region, subtropical Asia, and the Indo-Pakistan subcontinent but is now growing in many parts of the world such as Australia, USA, China, and Middle East countries [44]. This plant has potential toxic effect after ingestion. All parts of oleander are toxic containing oleandrin, oleandrigenin, and other cardiac glycosides [45]. Toxic exposure of humans and different species of domestic animals to *N. oleander *cardenolides occurs commonly throughout the geographic regions where this plant grows [46, 47]. N.O. extracts have significant dose- and time-dependent cytotoxic effects. Animals exposed to the plant are often found suddenly dead owing to cardiac dysfunction [48, 49]. The plant has been extensively studied both phytochemically, and pharmacologically and a number of compounds with variety of activities have been isolated, but the margin of safety is narrow [50]. 

The aim of this study was to investigate the hepatic damage with noninjured liver after induction of N.O. leaves extract in rats.

## 2. Materials and Methods

### 2.1. Animals

Male Wistar rats (about 200 g body weight) were arranged from the Department of Zoology, GCS (Lahore-Pakistan), kept under standard conditions with 12 h light/dark cycles and access to fresh water and food pellets *ad libitum*. All the animals were acclimatized under standard laboratory condition for a period of 2 weeks before the commencement of the experiment.

### 2.2. Materials

All chemicals were of analytical grade and obtained from commercial sources as indicated: Kits for the estimation of Iron parameters from Randox Laboratories, Ltd. (U.K), *hepcidin* ELISA kit Cat. No. CDN-T4096 from Creative Diagnostics (NY, USA), and Serum ferritin kit pack from Vitros Immunodiagnostics (Ortho-clinical diagnostics, Johnson and Johnson company, USA). All other reagents and chemicals were from Sigma-Aldrich Chemie (Munich, Germany) or Merck (Darmstadt, Germany).

### 2.3. Antibodies

A mouse anti-rat ectodysplasin-1 (ED1) monoclonal antibody from Serotec, ref no. MCA 341- Duesseldorf, Germany, was used as 1 : 100 dilution. Rabbit anti-mouse Horseradish peroxidase (HRP) conjugated from DAKO P0161 in 1 : 40 dilution was used as secondary antibody and described according to manufacturer's instructions. 

### 2.4. Experimental Design

N.O. leaves extract (10 mL/kg) was injected intramuscularly in both hind limbs using micropuncture needle (0.25 × 6 mm) of Wistar rats, and control animals received saline injection. The experimental protocol followed a minimally invasive procedure. All the animals were anesthetized and sacrificed after 3, 12, and 24 h with ketamine-distilled water mixture (1 : 1), (50 mg/mL of ketamine) i.p. Liver was excised, immediately after sacrifice, and rinsed with physiological sodium saline, and portion was fixed in 10% formalin for histological studies. Blood of the control and treated animals was drawn through cardiac puncture and processed for measurement of serum *hepcidin* and iron profile.

### 2.5. Processing for Serum Indices

Blood samples were allowed to clot overnight at 4°C and centrifuged for 20 min at 2000 g. Hemolysis-free serum samples were removed under sterile conditions, and indices were determined using ready to-use-Kits. Treated samples contained serum from N.O. treated rats at different time points mentioned above after the N.O. injection.

### 2.6. Estimation of Serum Iron

 Colorimetric method is used in which ferric iron is dissociated from its carrier protein, transferrin, in an acid medium and simultaneously reduced to the ferrous form. The ferrous iron is then complexed with the chromogen, a sensitive iron indicator, to produce a blue chromophore which absorbs maximally at 595 nm.

### 2.7. Estimation of Serum Ferritin

Quantification of the reactions was done employing fully automated chemistry analyzer ECiQ VITROS (Johnson and Johnson Company, USA) using the protocol provided by the manufacturer. For calibration of the instrument VITROS, immunodiagnostic ferritin calibrators were used. Briefly, ferritin present in the sample reacted simultaneously with a biotinylated antibody (sheep polyclonal anti-ferritin) and horseradish peroxidase (HRP)-labeled antibody conjugate (mouse monoclonal anti-ferritin). The amount of HRP conjugate bound was directly proportional to the concentration of ferritin present in the sample.

### 2.8. Enzyme-Linked Immunosorbent Assay

Blood samples were allowed to clot overnight at 4°C and centrifuged for 20 min at 2000 g. Serum was removed and stored in aliquots at −20°C. All reagents, samples, and working standards were brought to room temperature and prepared according to the manufacturer's directions. Quantification of the reactions was determined by the optical density using an automated ELISA reader (Biorad-680 Microplate reader, USA) at 450 nm. The magnitude of the absorbance for this developed color is proportional to the amount of *hepcidin*.

### 2.9. Histological Examination

The fixed tissue specimens were processed by standard methods and stained for Hematoxylin and Eosin (H & E) from Sigma-Aldrich using the protocol provided by the manufacturer.

### 2.10. Immunohistology

Immunohistochemical evaluation was performed on 4 *μ*m thin, formalin-fixed, paraffin embedded serial sections. Briefly, the paraffin-embedded sections were deparaffinized and rehydrated using graded alcohols to phosphate-buffered saline (PBS).The sections were incubated in a humidified chamber with the first antibody directed against ED1, diluted in PBS at 1 : 100 for 1 hour at room temperature. Negative controls were obtained by incubating with isotype-specific mouse/rabbit/goat IgGs instead of the specific primary antibody. After washing, the slides were covered with peroxidase-conjugated anti-rabbit/anti-mouse/anti-goat immunoglobulins preabsorbed with normal rat serum to avoid cross-reactivity. Slides were washed and incubated with PBS containing 3,3-diaminobenzidine (0.5 mg/mL) and H_2_O_2_ (0.01%) for 10 minutes to visualize immune complexes. Nuclei were counterstained with Meyer's hemalum solution before slides were mounted with coverslips. 

### 2.11. Statistical Analysis

The data were analyzed using Prism Graph pad 5 software (San Diego, CA). Statistical significance was calculated by one-way analysis of variance (ANOVA) and Dunnett post hoc test. Significance was accepted at *P* < 0.05. Results are shown as mean ± S.E.M. with *n* = 3.

## 3. Result

### 3.1. Changes in the Total Iron, Ferritin, and TIBC

**Figure 1:**
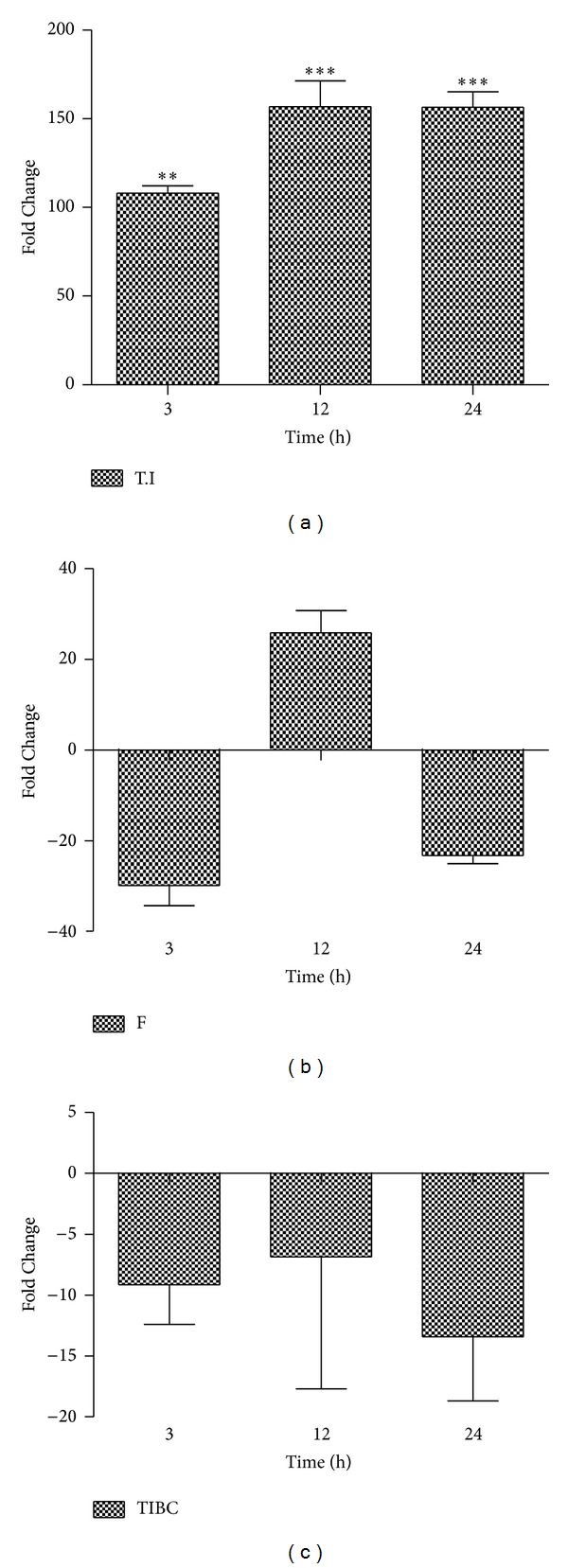
Serum iron profile levels in rats after N.O administration. (a) Total Iron (T.I) level continuously elevated after 3 h up to the studied time point. (b) Serum ferritin (F) showed increase only at 12 h with sudden decrease thereafter. (c) Total iron binding capacity (TIBC) levels in serum are shown with overall decline in the parameter, when compared to control values which were transformed to zero. These results are representative of three animal series (statistically significant at *P < 0.05; mean ± SEM).

Significant increment of total iron content in the serum was noted during the course of the study (*P* = 0.0001) with maximum increase of 156.87% after 12 h with single shot of N.O. leaves extract (*P* < 0.001). Approximately 100% rise was seen after 3 h, and almost more than 100% rise was observed thereafter by one-way ANOVA compared to control (Figure 1(a)). A negative change in serum ferritin was observed at 3 and 24 h of injection with approximately declines of 29 and 23%, respectively, but the changes were not significant (Figure 1(b)).

TIBC showed an overall decreasing trend for all the study time points with maximum decrease peaked at 24 h (Figure 1(c)).

### 3.2. *hepcidin* Levels in Serum

**Figure 2:**
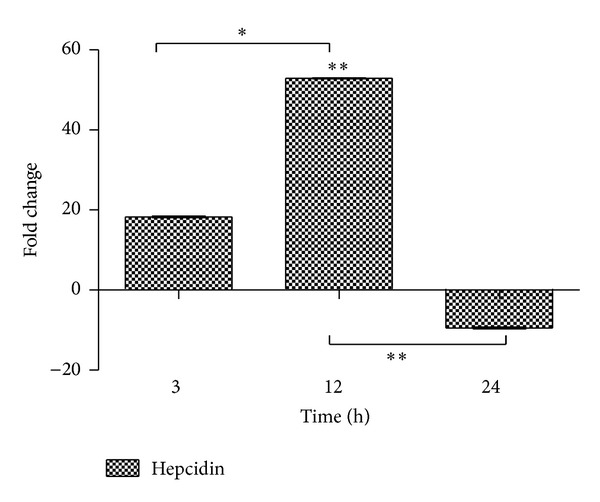
ELISA performed for the serum level of *hepcidin*. A time-dependent increase in serum *hepcidin* concentration was found up to 12 h with significant negative change in concentration thereafter (9.5-fold), when compared to control values which were transformed to zero (statistically significant at *P < 0.05; mean ± SEM).

An ELISA was performed to analyze serum *hepcidin* concentration. N.O. extract administration greatly increased serum *hepcidin* which reached the peak at 12 h (*P* < 0.01) compared with the control group while a decrease of 9.53% in value was noted after 24 h (Figure 2). 

### 3.3. H&E and Immunohistological Findings

Histopathological analysis of H&E and ED1 antibody stained liver tissue sections revealed that the administration of N.O. leaves extract (10 mL/kg) up to 24 h caused a disruption in general microarchitecture of the hepatocytes with predominant macrophages as seen by ED1 positive staining.

**Figure 3:**
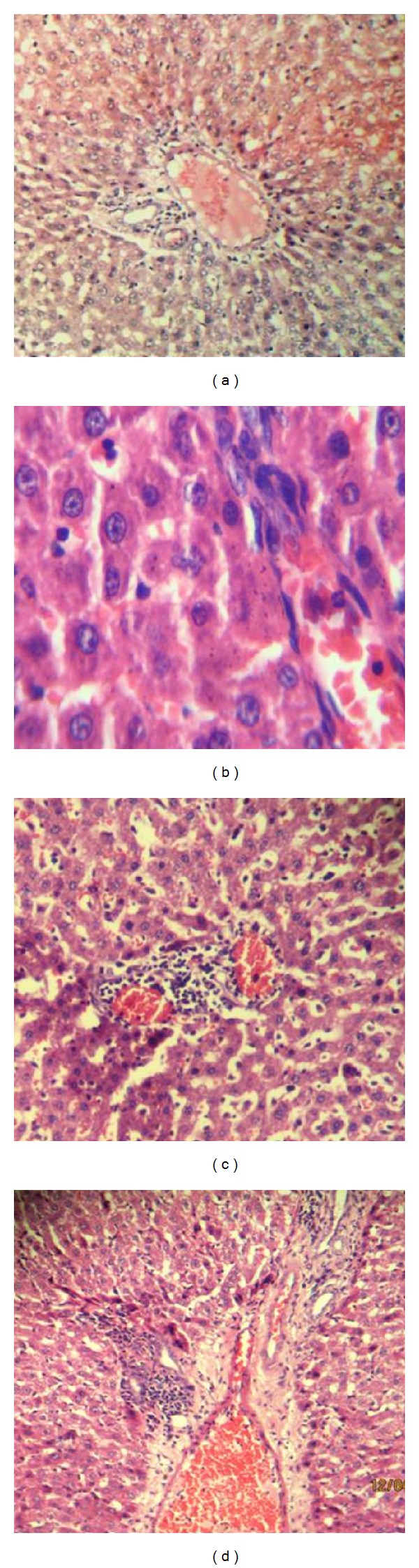
Hematoxylin-eosin stainings of liver sections of N.O. treated rat after 3- (b), 12- (c), and 24- (d) hour time courses of sterile muscle abscess compared with control (a). Treated sections showed a disruption in general microarchitecture of the hepatocytes with predominant macrophages observed throughout the studied time points.

**Figure 4:**
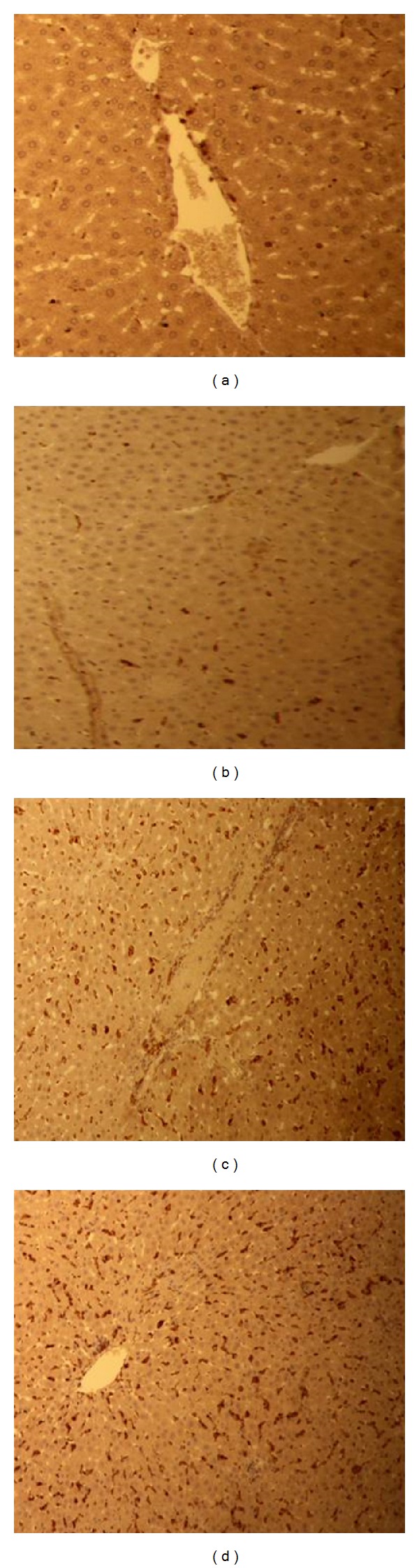
Immunohistology: sections of liver are stained with an antibody directed against ED1 followed by peroxidase staining. Progressive recruitment of ED1^+^ cells are seen after 3 (b), 12 (c), and 24 (d) hours of N.O. administration compared to control (a).

 Vacuolization of nucleoplasm consequently loss of polarity, loss of polarity with prominent nucleoli was observed after 3 h; however, sinusoidal spaces seemed to maintain their structure with very few ED1^+^ cells (Figures 3(b) and 4(b)). At 12 h, in H&E section, eccentric nuclei were observed with extravasations of leukocyte. Irregular nuclear features are indicative of N.O. toxicity (Figure 3(c)). Marked increase in the number of macrophages around the portal vessel and surrounding hepatocytes of the rat liver against ED1 antibody was observed (Figure 4(c)). The section (H&E) after 24 h of induction showed extensive congestion of hepatocytes with an irregular cytoplasm and disruption in typical lobular architecture with loss of polarity in the hepatic cells as compared to control (Figure 3(d)). ED1^+^ cells were recruited maximally at this study time point (Figure 4(d)). 

## 4. Discussion

In the current study, alterations in the level of *hepcidin* and iron profile including total iron (T.I), serum ferritin (F), and total iron binding capacity (TIBC) in serum together with histopathological changes in liver section against N.O. leaves extract through sterile muscle abscess (indirect liverinjury) were studied. N.O. extract was shown to have time-dependent effects and provided a model of acute liver damage in rats.

Iron is interwoven with many of the cellular functions including proliferation of cells and as a constituent of Heme and iron-sulfur proteins [38, 51, 52]. Under physiological conditions, the amount of absorbed iron is equivalent to compensate for daily iron loss due to the sloughing of epithelial cells, blood loss, and sweat. Therefore, cellular iron deficiency stops growth of cell and even might lead to its death. Homeostasis of iron is maintained by coordinated regulation of the rates of absorption, recycling, and mobilization of its stores [52]. 

Fenton reaction, a condition usually occurring during iron over load [53] is concurrent with our study. This might be due to either of oxidative stress or inflammation. Free iron not only produced free radicals which cause oxidation, but it also deposited in hepatocytes/Kupffer cells [54–56]. Inflammation caused by indirect liver injury due to N.O. extract observed in the present work resulted in an increased level of total serum iron throughout the course of study which contributes to the initiation and perpetuation of liver injury substantiated histological analysis. Supportive findings were observed with increased hepatic iron which contributes to alcohol toxicity of liver due to the production of reactive oxygen species [57]. Similarly, development of cirrhosis in mice was reported with the experimental addition of iron, supplemented with subtoxic dose of CCl_4_ [58]. In contrast, inhibition of liver fibrosis was noted through iron deficient diet which hampers oxidative stress, inflammation, and hepatic stellate cell activation [59] which indirectly supports the present study. The interpretation of an elevated serum ferritin after 12 h might also suggest the iron over load; acute inflammatory condition with a significant increase of hepcidin as well at the same study point supports the current findings.

*hepcidin*, a small cysteine-rich cationic peptide with antibacterial activity, secreted predominantly from hepatocytes [28–30] whose expression is regulated positively by body iron load/stores and inflammatory signals, chiefly IL-6, and suppressed by hypoxia and anemia [30, 38, 51, 52, 60]. Measurement of *hepcidin* concentrations can be used for the diagnosis of iron related disorders and could complement the most frequently used indicators of total body iron content, such as serum iron and ferritin. In the current study, an early rise of serum *hepcidin *was detected at 3 h which peaked at 12 h (53 fold) might be suggestive of inflammation, which is strongly related to increased *hepcidin* levels regardless of iron store and erythropoietic status [61]. A sharp decrease of 9.5 fold at a later time point (24 h) might reflect the beginning of necrosis of hepatocytes in response to the toxin present in the extract. Similar time-dependent increase in serum iron and pro*hepcidin* concentration was found in CCl_4_-induced liver injury in Wister rats [3].

Disturbance in any one of the *hepcidin* regulatory proteins, including the *HFE*, Tf receptor 2 (*TfR2*), hemojuvelin (*Hjv*), bone morphogenetic srotein 6 (*BMP6*), matriptase-2, neogenin, and transferrin (*Tf*) causes inappropriate regulation of its expression and consequently results in either iron overload or iron deficiency. Strong upregulation of *hepcidin* gene expression was also reported in the liver of iron-overloaded mice [30] as evident in the current study. 

H&E and ED1 antibody stained liver tissue sections showed a disruption in general microarchitecture of the hepatocytes with predominant macrophages observed throughout the studied time points. Recruitment of inflammatory cells observed in the present study may be due to APR following injury against the toxic effects of N.O. leaves extract. Infiltration of mononuclear inflammatory cells into the portal space with scattered necrosis of hepatocytes in H&E stained sections was registered with an oral administration of dried oleander leaves (110 mg/kg) [46, 62]. Maximum recruitment of ED1^+^ cells was noted after 24 h of N.O. administration. Similar results were found with maximum recruitment of ED1^+^ cells to the site of injury after 24 h of intramuscular TO-injection in rats [63].

## 5. Conclusion 

In conclusion, sterile muscle abscess with ethnobotanical products like N.O. was shown to have time-dependent effects and may induce APR resulting in change of serum *hepcidin*, ferritin, and total iron levels as well as hepatic damage which is evident with the recruitment of inflammatory cells into the organ and serves as a model of acute liver damage in rats.
